# CHRONIC OBSTRUCTIVE PULMONARY DISEASE SELF-MANAGEMENT USING TECHNOLOGY IN DAILY LIFE: A PILOT STUDY

**DOI:** 10.1093/geroni/igae098.3008

**Published:** 2024-12-31

**Authors:** Seoyoon Woo, Anastasiya Ferrell, Jamy Chulak

**Affiliations:** University of North Carolina Wilmington, Wilmington, North Carolina, United States; University of North Carolina Wilmington, Wilmington, North Carolina, United States; University of North Carolina Wilmington, Wilmington, North Carolina, United States

## Abstract

**Rationale:**

Self-management is crucial to preventing lung function decline and improving the quality of life but is rarely performed daily in people with chronic obstructive pulmonary disease (COPD). During the COVID-19, they were forced to use telemedicine and telerehabilitation for support. However, the extent to which such technology is used to manage COPD remains unknown. This study aimed to examine the use of technology for COPD self-management and its barriers and facilitators.

**Methods:**

A cross-sectional study using an online survey method was conducted. People with COPD(N=102; mean age=44years) completed a set of questionnaires, including the Self-Management Assessment Scale and the Media and Technology Usage and Attitudes Scale, and open-ended questions. Descriptive statistics, multiple regressions, and the interpretative thematic approach were used to analyze the data.

**Results:**

Compared to mobile device usage(94.2%), only 3.9% used it to self-manage COPD. However, over 60% expressed a willingness to use mobile apps for self-management. eHealth literacy significantly predicted both Media and Technology Usage(β=0.70,p=0.001) and Attitudes(β=0.20,p< 0.001). COPD self-efficacy significantly predicted all five domains of self-management needs: knowledge(β=0.33,p=0.003), goals for future(β=0.36,p=0.02), daily routines(β=03,p=0.004), emotional adjustment(β=0.53,p< 0.001), and social support(β=0.51,p=0.02). Barriers to using mobile apps included their reliability, forgetfulness, and usability, just to name a few. Mobile apps for COPD self-management can be more attractive if they feature monitoring and recording of symptoms and provide relevant resources.

**Conclusion:**

This study identified factors influencing the use of technology for COPD self-management. The findings can be considered in designing self-management intervention tools to help better manage COPD.

